# Demographic Characteristics and Survival in Young-Onset Colorectal Neuroendocrine Neoplasms

**DOI:** 10.3390/biomedicines12102411

**Published:** 2024-10-21

**Authors:** Deepak Vadehra, Sahithi Sonti, Beas Siromoni, Mrinalini Ramesh, Debduti Mukhopadhyay, Adrienne Groman, Renuka Iyer, Sarbajit Mukherjee

**Affiliations:** 1 Department of Medicine, Roswell Park Comprehensive Cancer Center, Buffalo, NY 14263, USA; deepak.vadehra@roswellpark.org (D.V.); sahithi.sonti@gmail.com (S.S.); renuka.iyer@roswellpark.org (R.I.); 2 School of Health Sciences, University of South Dakota, Vermillion, SD 57069, USA; beassiromoni@gmail.com; 3 Department of Internal Medicine, University at Buffalo, Buffalo, NY 14204, USA; mramesh3@buffalo.edu (M.R.); debdutim@buffalo.edu (D.M.); 4 Department of Biostatistics and Bioinformatics, Roswell Park Comprehensive Cancer Center, Buffalo, NY 14263, USA; adrienne.groman@roswellpark.org

**Keywords:** young-onset neuroendocrine neoplasms, young-onset adenocarcinoma, young-onset colorectal cancer (YOCRC)

## Abstract

Background/Objectives: Recent epidemiological studies have revealed an upward trend in young-onset colorectal cancer (YOCRC) overall, whereas specific data on young-onset colorectal neuroendocrine neoplasms (YONEN) remain limited. This study investigated the demographic characteristics and survival trends in YONEN and compared these with those of young-onset colorectal adenocarcinoma (YOADC), the most common histologic subtype of YOCRC. Methods: A retrospective analysis was conducted from 2000 to 2019 using the Surveillance, Epidemiology, and End Results (SEER) database. Survival outcomes were assessed using univariate and multivariable Cox proportional models, with demographic differences evaluated via Wilcoxon rank sum and Chi-square tests. Results: Out of 61,705 patients aged 20–49 with colorectal cancer, 8% had NEN, and 92% had adenocarcinoma. The YONEN cohort had a higher proportion of Black patients and a lower proportion of White patients than the YOADC cohort (21% vs. 13% and 44% vs. 57%, respectively). NEN was more commonly found in the rectum (79%), and adenocarcinoma was mostly colonic (57%) in origin. YONEN patients had better survival than YOADC patients. Multivariate analysis in YONEN patients revealed that Hispanic patients had better overall survival compared to White patients (HR 0.67, 95% CI 0.47–0.95, *p* = 0.024). Conclusions: Racial disparities should be investigated further to aid in policymaking and targeted interventions.

## 1. Introduction

Colorectal cancer (CRC) is the second leading cause of cancer-related death in the United States, with adenocarcinoma comprising the majority. According to the American Cancer Society, an estimated 52,550 individuals will succumb to CRC in 2023 [1]. A recent study by Tan et al. examined the mortality trends in colorectal cancer in the US [2]. The study revealed that the incidence rates in CRC from 1999 to 2020 decreased significantly from 26.42 to 15.98 per 100,000 individuals, with an Average Annual Percent Change (AAPC) of −2.41. However, the Age-Adjusted Mortality Rate (AAMR) of rectosigmoid cancer went up from 0.82 to 1.08 per 100,000 individuals, with an AAPC of +1.10. Males and Black patients had the highest AAMRs, with rates of 23.90 and 26.93 per 100,000 individuals, respectively. Moreover, the overall AAMR of CRC decreased for those aged 50 years and older but worsened from 1.02 to 1.58 per 100,000 individuals for YOCRC patients, with an AAPC of +0.75. These results show that disparities in CRC mortality persist across age, sex, race, geographic region, and urbanization level, underscoring the necessity for targeted public health interventions.

A study which investigated the impact of race in receiving guideline-concordant care for young-onset colorectal cancer (YOCRC) in the United States revealed significant findings [3]. Black patients with YOCRC were more likely to be deprived of surgery (adjusted odds ratio [aOR] 1.15, 95% confidence interval [CI] 1.07 to 1.24), have standard (less than 12) lymph nodes examined (aOR 1.11, 95% CI 1.05 to 1.17), and not receive chemotherapy (aOR 1.22, 95% CI 1.17 to 1.27) compared to Caucasian patients. Black patients with rectal cancer were more likely not to have complete staging (aOR 1.90, 95% CI 1.77 to 2.04), not undergo surgery (aOR 1.38, 95% CI 1.30 to 1.45) or chemotherapy (aOR 1.68, 95% CI 1.56 to 1.82), not start radiotherapy (aOR 1.20, 95% CI 1.14 to 1.27), not finish radiotherapy (aOR 1.20, 95% CI 1.12 to 1.30), and be given treatment in the incorrect order (aOR 1.25, 95% CI 1.16 to 1.34).

The projected figures for young-onset colorectal cancer (YOCRC) reveal a troubling pattern within the 20 to 49-year-old age bracket. By 2040, colorectal cancer is anticipated to rank as the second most prevalent cancer in this demographic due to its increasing incidence [4]. Significantly, instances and fatalities related to colorectal cancer in younger adults have shown an upward trajectory over the past decade and are projected to continue increasing over the next two decades. This concerning trend may be attributed to factors such as sedentary lifestyles, poor dietary habits, obesity, and a lack of routine screening in this age group [4]. These forecasts emphasize the critical need for heightened awareness, early detection, and screening programs to tackle the escalating burden of colorectal cancer among younger cohorts.

From a histological perspective, adenocarcinoma is the most common subtype of CRC, followed by neuroendocrine neoplasms (NEN), which are a rare subgroup of young-onset colorectal cancers [5]. According to the WHO system, the grades for neuroendocrine neoplasms (NEN) include grade 1, grade 2, grade 3 which are distinguished by the mitotic rate and Ki-67 indices [6]. Tumors are further divided into neuroendocrine carcinomas (NEC), which are high-grade with further subdivision into multiple categories. One of these categories includes mixed adenoneuroendocrine carcinomas (MaNEC). Mixed neuroendocrine non-neuroendocrine neoplasms (MiNEN), on the other hand, are a rare group of NENs that consist of a neuroendocrine and a non-neuroendocrine component, both exceeding 30%. They can be either well or poorly differentiated and were included as a separate category of NENs in the 2019 WHO classification [6]. A recent study by Abboud et al. showed that the rise in YOCRC may be attributed to the fact that the incidence of colorectal NENs is increasing at a rate even faster than adenocarcinoma in the young population [7]. It was observed that there has been a substantial increase in the incidence of neuroendocrine neoplasms compared to adenocarcinomas (ADC) in this population. Specifically, the incidence of NENs showed a much more significant rise than that of ADC, with an average annual percentage change (AAPC) of 2.65 for NENs compared to 0.91 for ADC. This difference in AAPC between NENs and ADC was statistically significant (*p* = 0.01), indicating a notable disparity in the trends of these histopathological subtypes. These findings underscore the necessity for increased awareness and targeted screening strategies to address the rising incidence of colorectal cancer, particularly neuroendocrine neoplasms, in the younger population, ultimately aiming to enhance early detection and improve patient outcomes.

Similarly, Lumsdaine et al. conducted a population-based study from 1992 to 2015 that identified a substantial rise in young-onset colorectal cancer (CRC), specifically rectal neuroendocrine neoplasms (NENs) [8]. The incidence of rectal NENs exhibited a significant increase across all age groups, particularly notable in individuals aged 45–54 and those over 55 years. In the younger age brackets of 20 to 44 and 45 to 54 years, the annual percent changes (APCs) for rectal NENs were calculated at 2.9 and 6.1, respectively, indicating a notable upward trajectory in incidence rates. Notably, the surge in rectal NENs played a substantial role in the overall increase in rectal cancer cases, with statistics revealing that NENs contributed significantly, accounting for 26.74% and 53.47% of the total increase in the respective age groups. These findings underscore the increasing impact of rectal NENs on the prevalence of young-onset colorectal cancer, underscoring the necessity for further research and clinical focus to address this emerging trend and its implications on patient care and management strategies.

Given the rarity of NENs and their typically slow progression, limited data exist concerning these tumors in younger individuals. Therefore, there is an unmet need to learn about overall survival (OS), disease-specific survival (DSS), and the factors affecting survival in young-onset colorectal NEN. This study aimed to explore patterns and disparities in survival rates among young individuals diagnosed with colorectal NEN and compare them with young-onset colorectal adenocarcinoma patients as well as those with average-onset colorectal NEN patients. Additionally, we aimed to identify factors affecting survival in colorectal NEN.

## 2. Materials and Methods

### 2.1. Study Design and Population

We conducted a retrospective study on YOCRC patients in the US between 2000 and 2019 using the Surveillance, Epidemiology, and End Results (SEER version 8.4.3 software, NIH/NCI, Bethesda, MD, USA) database. The SEER database compiles cancer-specific incidence data from population-based registries, covering approximately 35% of the US population [9]. Patients aged < 50 years were included. For the purpose of our analysis, we included adenocarcinomas, neuroendocrine tumors, neuroendocrine carcinomas, MiNEN, and MANEC. Patients with unknown stage, unknown grade, and unknown race were included in the model as they are SEER reportable statuses. Variables noted previously that could not be estimated were removed from the model. Our primary endpoint was to estimate overall survival (OS), defined as the time from diagnosis to death from any cause. Our secondary endpoint was disease-specific survival (DSS), defined as the time from diagnosis to death specifically due to cancer.

### 2.2. Covariates

Key variables of interest included the patient’s demographic characteristics such as age, race, sex, and disease characteristics. Clinical variables of interest included disease stage, grade, surgery, primary cancer site, and year of diagnosis. Race was categorized into non-Hispanic Whites, non-Hispanic Blacks, Hispanics, Asian Americans, and Native Americans. The stage was classified according to the AJCC Classification System [10] as Stage 0, 1, 2, 3, and 4. Grade was classified as 1, 2, 3, and undifferentiated. The primary cancer site was categorized as colon, rectum, or rectosigmoid, and the year of diagnosis was divided into five periods: 2000–2003, 2004–2007, 2008–2011, 2012–2015, and 2016–2019.

### 2.3. Statistical Analysis

Demographic and clinical characteristics of the overall sample were summarized. For categorical variables, frequencies and relative frequencies were provided and compared using the Pearson chi-square test while contrasting YONENs and YOADCs. The Kruskal–Wallis test, a non-parametric test, was used for ordinal or continuous variables. The effects of various factors on OS and DSS were estimated using Cox proportional hazard models adjusted for age at diagnosis (20–39, 40–49, ≥50), sex, race, stage, grade, primary site, tumor size, surgery, and year of diagnosis. Result estimates were expressed as hazard ratios (HR) with 95% confidence intervals (CI). The Kaplan–Meier method calculated the OS and DSS. Yearly mortality rates per 100,000 were calculated to examine differences among histology and age groups. Unknown stage, unknown grade, and unknown race were included in the model as they are SEER reportable statuses. Variables that could not be estimated, were removed from the model. All model assumptions, proportional hazards and goodness of fit were evaluated visually using standard residual plots (Schoenfeld residuals versus time and standardized residuals versus predicted values). The significance level was denoted by *p* < 0.05. SAS, version 9.4 statistical software (SAS Institute Inc., Cary, NC, USA) was used for all statistical analyses.

## 3. Results

There were 61,705 patients in the young-onset colorectal cancer (YOCRC) group, of which 5128 belonged to the young-onset neuroendocrine neoplasms (YONEN) cohort, and the remaining 56,577 cases were part of the young-onset adenocarcinoma (YOADC) group. Fifty-two percent of the YONEN group were female, compared with 45.8% in the YOADC group. In the YONEN group, 43.6% were White, 20.6% were Black, 18.2% were Hispanic, 11.8% were Asian, and 1.2% were Native American. In the YOADC group, 58% were White, 17.6% Hispanic, 13.15% Black, 10.3% Asian, and 1% Native American. The primary disease site for most YONENs was the rectum (78.7%), followed by the colon (17.3%) and the rectosigmoid region (4%). Conversely, the majority of YOADCs had the colon as their primary disease site (61.2%), with 28.3% in the rectum and 10.5% in the rectosigmoid. The most common stage at diagnosis for YONENs was Stage I (21%), followed by 6.3% with Stage IV disease. However, a significant majority (67.2%) did not have a reported stage in the SEER database. For the YOADC group, 29% had Stage III disease at diagnosis, another 23.6% had Stage IV disease, with only 14.8% not having a reported stage. The demographic characteristics are described in Table 1.

We also compared young and average-onset NEN patients. We found that of patients from the younger group (<50 years), 52% of the group were female patients, compared with 48.9% in the average-onset group (patients aged 50 and above). Hispanic patients were overrepresented in the younger age group (18.2%), compared to the average-onset group (13.5%). YONEN patients had a higher proportion of rectal tumors compared to the average-onset NEN patients (79% vs. 69%). Patients in the average-onset group were also found to have a significantly higher proportion of grade III tumors (8%) compared to YONEN patients (4.3%), *p* < 0.001. This comparison is detailed in Table 2.

**Figure A1:**
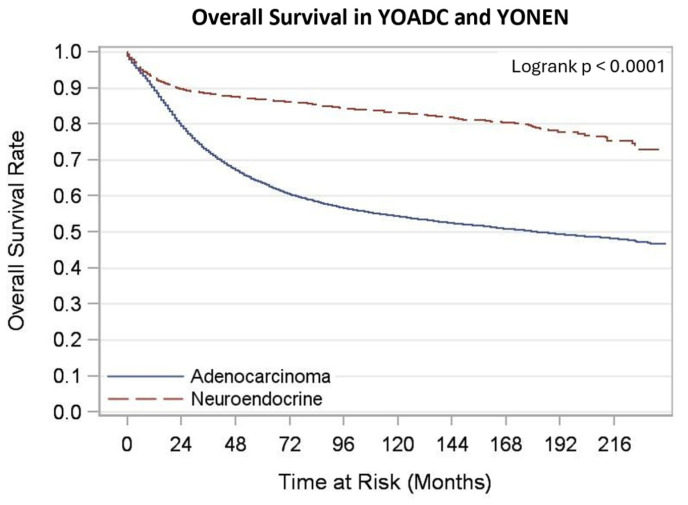
OS rates in YOADC and YONEN patients (N = 45,827).

**Figure A2:**
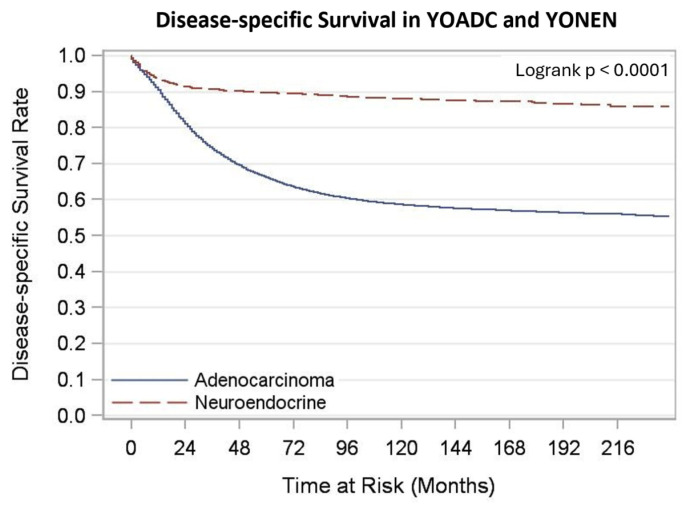
DSS rates in YOADC and YONEN patients (N = 45,827).

**Figure A3:**
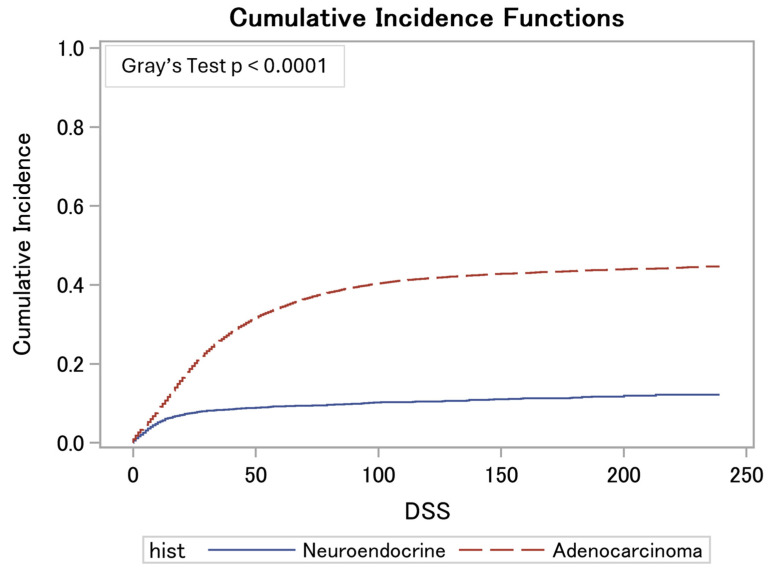
Cumulative incidence function and Fine–Gray model regarding DSS for YONEN versus YOADC.

We analyzed overall survival and disease-specific survival for YONEN and YOADC patients. YONENs had a 1-year survival rate of 0.94 (95% CI: 0.93, 0.94) and a 5-year survival rate of 0.88 (95% CI: 0.87, 0.89), whereas YOADCs had a 1-year survival rate of 0.90 (95% CI: 0.90, 0.90) and a 5-year survival rate of 0.63 (95% CI: 0.63, 0.64) with a median follow up time of 105 months. The 1-year disease-specific survival (DSS) rate for YONENs was 0.95 (95% CI: 0.94, 0.95) compared to 0.91 (95% CI: 0.91, 0.91) for YOADCs. The 5-year DSS rate for YONENs was 0.91 (95% CI: 0.90, 0.92) versus 0.66 (95% CI: 0.65, 0.66) for YOADCs with a median follow up time of 97 months. Survival rates by age group are detailed in Table 3 as well as the Kaplan–Meier curves in Figure A1 and Figure A2. Cumulative incidence function and the Fine–Gray model were also performed, and the factors found to be significantly associated with DSS in the Cox regression were also retained in the Fine–Gray model (Figure A3).

**Figure 1:**
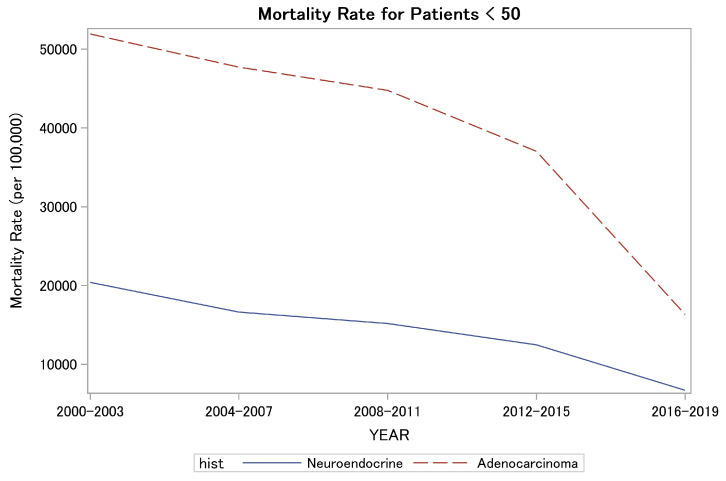
Mortality rates in YOADC and YONEN in the last two decades (per 100,000).

Next, we examined the mortality trends. The rate of deaths per 100,000 decreased from 49,554 in 2000–2003 to 15,527 in the period between 2016 and 2019. Despite the rising incidence, there was a numerical improvement in mortality rates in recent years; the YONEN mortality rate decreased from 20,403 per 100,000 in 2000–2003 to 6705.3 per 100,000 in 2016–2019 (Figure 1). A similar magnitude of decrease was observed in the YOADC group, from 51,928.6 per 100,000 in 2000–2003 to 16,331.9 in 2016–2019 (*p* < 0.001) (Figure 1).

We also examined survival differences between the young-onset (<50) and average-onset (≥50) colorectal neuroendocrine neoplasms cohorts and found that the overall survival in the young-onset cohort was better than the average-onset NEN cohort. The 5-year overall survival rate between the young-onset and average-onset groups was 0.89 (95% CI: 0.88, 0.89) versus 0.65 (95% CI: 0.64, 0.66) after a median follow up time of 96 months. Similarly, the 5-year disease-specific survival rate for the young- versus average-onset groups was 0.92 (95% CI: 0.92, 0.92) versus 0.76 (95% CI: 0.75, 0.77) after a median follow up time of 82 months. This is likely related to the fact that younger patients have fewer comorbidities and better functional status, and hence can tolerate more aggressive treatments, leading to better survival rates. Survival rates of the young-onset versus average-onset colorectal NEN are detailed in Table 4.

Finally, we assessed factors affecting overall survival and disease-specific survival in the YONEN population. Higher grade was associated with worse overall survival (OS) (Grade III vs. Grade I; HR = 11.88, *p* < 0.001 [RMST Grade III: −1.2, CI −1.65, −0.75, *p* < 0.001] and Undifferentiated vs. Grade I; HR = 11.23, *p* < 0.001 [RMST Undifferentiated: −1.65, CI −2.5, −0.79, *p* < 0.001]). The Hispanic race was also found to be associated with improved overall survival (HR = 0.67, *p* = 0.024 and RMST Hispanic: 0.042, CI 0.009, 0.074, *p* = 0.013). See Table A1 and Table A2. Restricted mean survival time regression was applied on both OS and DSS, see Table A3 and Table A4.

## 4. Discussion

The increase in colorectal cancer in young people is attributable to the rise in both neuroendocrine neoplasms and adenocarcinomas. However, Abboud et al. [7] have shown that the rate of increase in neuroendocrine neoplasms in the population under 50 years is significantly higher than that of adenocarcinoma. Adolescents and young adults (AYA) represent a unique population up to age 39 years of age. These patients are distinctive, as rare cancers are overrepresented in this group [11,12]. Given that this population does not routinely undergo screening colonoscopies, we wanted to see the distribution pattern of adenocarcinoma versus neuroendocrine neoplasms in these patients compared to those in the 40-49 age group. Similar to Abboud et al. [7], we noted that the AYA population is overrepresented with NEN compared to adenocarcinomas (Table 1).

We also noted several demographic differences between the YONEN and average-onset neuroendocrine neoplasm populations. When we compared these two groups, we found that female and Hispanic patients were overrepresented in the younger population and that the primary tumor site was more likely to be in the rectum. This calls for targeted interventions in younger female and Hispanic patients. Moreover, since these tumors are commonly found in the rectum, screening via flexible sigmoidoscopy should be considered in yearly testing.

A considerably larger fraction of YONEN patients were Black when compared to those with adenocarcinoma (20.6% vs. 13.1%) (*p* < 0.001). This is consistent with what has been seen in other studies. In a SEER analysis [13] of all NENs, it was seen that Black patients had a higher incidence and worse survival when compared to other races. However, our multivariate analysis did not identify Black race as an individual prognostic factor. This could be due to relatively small sample size in our study. In another study, Herring et al. showed significant differences in gene expression between Black and White pancreatic NEN (pNEN) patients, indicating potential disparities in tumor microenvironment that could affect outcomes [14]. RNA sequencing of pNENs from Black and White patients identified 372 markedly differentially expressed genes and 179 enriched gene sets, with key pathways associated with angiogenesis, blood vessel formation, cell migration, and immune response. Black patients showed enrichment in gene sets associated with blood vessel formation and cellular migration, while immune response pathways were downregulated in this group. These findings suggest distinct tumor biology in NENs from Black patients that may contribute to the disparate outcomes observed in this population, highlighting the importance of further validation and consideration of genetic ancestry in future studies.

Another critical difference we saw was the primary site of the tumor: 78.7% of YONENs were in the rectum, whereas 61.2% of YOADCs were primarily in the colon. Interestingly, we noted essential survival differences in the YONEN vs. YOADC population. Despite the rapid increase in the incidence of NENs, the median overall survival was high when compared to YOADCs. We looked at the overall survival rate in YONENs in the adolescent and young adult (AYA, 20–39 years) and 40–49-year-old subgroups. The age-specific OS rate slightly worsened with increasing age (0.96 vs. 0.93 1-year survival rate and 0.91 vs. 0.87 5-year survival rate, respectively). Nevertheless, this trend was not seen in the YOADCs (0.90 vs. 0.90 for a 1-year survival rate and 0.62 vs. 0.64 for a 5-year survival rate). It may be hypothesized that while the rise in incidence in YOCRC is attributable to the exponential increase in YONENs, the poor OS in this population is primarily still due to YOADCs, suggesting that adenocarcinoma in the young-onset cohort, specifically in the AYA cohort is an aggressive subtype [15,16].

Even with the differences in overall survival, mortality rates for both YOADC and YONENs consistently decreased over the last two decades in our study. This is in line with the decrease in mortality in all NENs, seen in a SEER analysis from 2017 [17] and an improved OS in the YOADC population seen in an NCDB analysis conducted in 2021 [18]. This may be attributable to multiple factors such as earlier screening and more treatment options in our therapeutic armamentarium. Research into available therapies is moving at a fast pace, with developments such as the tremendous success of PRRT [19,20].

Finally, we looked at the potential factors affecting mortality in the YONEN population and, predictably, found that the higher grade was associated with worse overall survival. We also found that Hispanic patients had better outcomes than White patients, corroborating the existing literature. A SEER analysis studying racial/ethnic disparities in non-pancreatic NENs found that Hispanic patients had the best overall survival when compared with non-Hispanic White and Black patients [21]. The study by Gosku et al. uncovered notable disparities in survival outcomes across racial and ethnic groups, shedding light on the nuanced impact of race and ethnicity on disease prognosis. Hispanic patients emerged as a cohort with distinct survival advantages, showcasing better overall survival rates than non-Hispanic White patients, with a hazard ratio of 0.89 (0.81–0.97). This lower risk of mortality among Hispanic individuals underscores a significant disparity in outcomes that warrants further investigation. Moreover, when examining specific primary tumor sites, Hispanic patients demonstrated superior overall survival in locations such as the small intestine and rectum, with hazard ratios of 0.81 (0.69–0.96) and 0.79 (0.63–0.99), respectively [20]. These findings suggest a potential biological or treatment-related advantage for Hispanic patients in these particular anatomical sites, highlighting the complexity of factors influencing survival disparities in neuroendocrine neoplasms.

The improved survival rates of Hispanics/Latinos compared to non-Hispanic Whites can be attributed to a complex interplay of genetic, behavioral, cultural, and environmental factors [22]. Genetic variances across racial/ethnic groups may influence survival outcomes. Behavioral differences, such as smoking patterns, also contribute, with Hispanics/Latinos potentially engaging in behaviors that confer a survival advantage. Despite often having lower socioeconomic status, this group may experience similar or better health outcomes, suggesting the influence of other factors. Cultural aspects like *familismo*, a strong family-oriented philosophy prevalent in Hispanic culture, could play a crucial role in promoting better health outcomes [22]. Additionally, survival advantages may arise not just from differences in healthcare access, but also from disparities in environmental exposures, cultural influences, and treatment approaches across racial/ethnic groups [22]. These elements together underscore the multifaceted reasons behind the superior survival outcomes observed in this demographic. Nevertheless, our findings highlight the critical role of race and ethnicity as independent prognostic factors in neuroendocrine neoplasms, emphasizing the importance of tailored interventions and personalized treatment strategies to address disparities and improve overall survival rates for diverse patient populations.

In moving forward from the findings of this study on young-onset colorectal neuroendocrine neoplasms, several key areas warrant further investigation to advance our understanding and improve patient outcomes. Better documentation at diagnosis of stage would aid in identifying differences in stage at presentation if any. Investigating genetic markers could lead to personalized treatment approaches, while examining the impact of lifestyle factors such as diet, exercise, and environmental exposures could provide valuable insights into disease development and progression. In addition, there are data showing that higher pain and stress scores using validated scales can correlate with poor outcomes in other diseases and the impact of these markers warrants further study in this group where anxiety and stress are expected to be high, underscoring the importance of databases that capture these measures [23]. Evaluating novel treatment modalities, including immunotherapy and targeted therapies, through randomized clinical trials, will help identify optimal strategies for YONEN patients.

Long-term follow-up studies are essential to assess survival outcomes and quality of life, helping to optimize patient care over time. Additionally, research on the impact of health insurance and care disparities is pivotal for addressing inequities, especially among minority groups [24,25]. Prior studies have documented disparities in relative survival that often impact minority groups [26]. Investigations have found that minority patients often have inadequate insurance that in turn results in increased risk of locally advanced disease on diagnosis [27]. Investigating barriers to healthcare access and designing interventions to reduce these disparities can improve outcomes [28]. Healthcare policy analysis should focus on evaluating existing policies and advocating for targeted interventions to reduce treatment disparities [29]. Implementing strategies like mobile screening programs and outreach oncology clinics can serve medically underserved communities, advancing health equity [30,31]. These research directions aim to tailor interventions to individual needs and enhance the quality of care and survival rates for YONEN patients.

Our study is not without limitations. Firstly, the study’s retrospective design may introduce biases and limitations in data collection, analysis, and interpretation. For example, detection bias or misclassification bias could have impacted our survival outcomes. Secondly, we utilized data from the Surveillance, Epidemiology, and End Results (SEER) database, which may have limitations in terms of data accuracy, completeness, and consistency. The quality of the data in the SEER database, especially the significant amount of unreported data on disease stage and grade, might have impacted some of the study’s findings like the multivariate analysis. Furthermore, the grades reported in the SEER database are not concordant with the WHO grading classification of NENs, with the database reporting Stage IV NEN [32]. Moreover, the classification of NETs has changed several times during the study period. We also removed the variables that could not be estimated from the multivariate model. Finally, the study may not have accounted for all potential confounding variables that could influence the outcomes of interest, such as comorbidities, or treatment variations like time to treatment initiation, quality of surgery, or type of chemotherapy, which were not available in the SEER database. Despite these limitations, our study was one of the largest studies to look at the demographic characteristics and survival of YONEN patients at a population level.

## 5. Conclusions

In conclusion, our study identified several sociodemographic disparities in YONEN and YOADC patients. We also found that despite increasing incidence rates, mortality rates have been steadily decreasing in this population. Further research is needed to understand the disparities for resource allocation as well as implementation of appropriate healthcare policies.

## Figures and Tables

**Table 1:** Demographic and clinical characteristics of young-onset colorectal neuroendocrine neoplasms and adenocarcinomas.

		Neuroendocrine Neoplasms	Adenocarcinoma
Age	20–39	1714 (33.4%)	14,164 (25%)
	40–49	3414 (66.6%)	42,413 (75%)
Sex	Male	2456 (47.9%)	33,108 (53.7%)
	Female	2672 (52.1%)	28,597 (46.3%)
Race	White	2235 (43.6%)	32,525 (57.5%)
	Black	1054 (20.6%)	7429 (13.1%)
	Asian	603 (11.8%)	5830 (10.3%)
	Native American	61 (1.2%)	573 (1.0%)
	Hispanic	935 (18.2%)	9937 (17.6%)
	Not reported	240 (4.7%)	283 (0.5%)
Year of diagnosis	2000–2003	794 (15.5%)	9748 (17.2%)
	2004–2007	950 (18.5%)	10,911 (19.3%)
	2008–2011	1054 (20.6%)	11,129 (19.7%)
	2012–2015	1122 (21.9%)	11,545 (20.4%)
	2016–2019	1208 (23.6%)	13,244 (23.4%)
Disease Site	Colon	887 (17.3%)	34,614 (61.2%)
	Rectosigmoid	204 (4.0%)	5954 (10.5%)
	Rectum	4037 (78.7%)	16,009 (28.3%)
Disease Grade	I	1760 (34.3%)	3848 (6.8%)
	II	302 (5.9%)	36,234 (64%)
	III	220 (4.3%)	9405 (16.6%)
	Undifferentiated	112 (2.2%)	971 (1.7%)
	Not reported	2734 (53.3%)	6119 (10.8%)
Overall Stage	I	1076 (21.0%)	6873 (12.1%)
	II	102 (2.0%)	10,695 (18.9%)
	III	129 (2.5%)	16,526 (29.2%)
	IV	323 (6.3%)	13,349 (23.6%)
	Not reported	3448 (67.2%)	8372 (14.8%)

**Table 2:** Colorectal neuroendocrine neoplasms: descriptive statistics by age (<50 vs. ≥50).

		<50	≥50	*p*-Value
Sex	Male	2456 (47.9%)	10,648 (51.1%)	*p* < 0.001
	Female	2672 (52.1%)	10,209 (48.9%)	
Race	White	2235 (43.6%)	10,420 (50.0%)	*p* < 0.001
	Black	1054 (20.6%)	4214 (20.2%)	
	Asian	603 (11.8%)	2490 (11.9%)	
	Native American	61 (1.2%)	158 (0.8%)	
	Hispanic	935 (18.2%)	2825 (13.5%)	
	Not reported	240 (4.7%)	750 (3.6%)	
Year of diagnosis	2000–2003	794 (15.5%)	2773 (13.3%)	*p* < 0.001
	2004–2007	950 (18.5%)	3579 (17.2%)	
	2008–2011	1054 (20.6%)	4428 (21.2%)	
	2012–2015	1122 (21.9%)	4931 (23.6%)	
	2016–2019	1208 (23.6%)	5146 (24.7%)	
Disease Site	Colon	887 (17.3%)	5668 (27.2%)	*p* < 0.001
	Rectosigmoid	204 (4.0%)	864 (4.1%)	
	Rectum	4037 (78.7%)	14,325 (68.7%)	
Disease Grade	I	1760 (34.3%)	6973 (33.4%)	*p* < 0.001
	II	302 (5.9%)	1272 (6.1%)	
	III	220 (4.3%)	1678 (8.0%)	
	Undifferentiated	112 (2.2%)	684 (3.3%)	
	Not reported	2734 (53.3%)	10,250 (49.1%)	
Overall Stage	In situ	50 (1.0%)	192 (0.9%)	*p* < 0.001
	I	1076 (21.0%)	3945 (18.9%)	
	II	102 (2.0%)	473 (2.3%)	
	III	129 (2.5%)	1080 (5.2%)	
	IV	323 (6.3%)	1760 (8.4%)	
	Not reported	3448 (67.2%)	13,407 (64.3%)	
Tumor size in mm	Mean/StdErr	15.1/0. 5	20.3/0.4	*p* < 0.001

**Table 3:** Age-specific OS and DSS rates at 1 year and 5 years in YONEN and YOADC patients.

		Age Group (Years)	
		20–39	40–49
Overall Survival	1-year survival rate (NEN) (95% CI) (N = 5128)	0.96 (0.94, 0.96)	0.93 (0.92, 0.94)
	5-years survival rate (NEN) (95% CI) (N = 5128)	0.91 (0.89, 0.92)	0.87 (0.86, 0.88)
	1-year survival rate (ADC) (95% CI) (N = 56,577)	0.90 (0.89, 0.90)	0.90 (0.90, 0.90)
	5-years survival rate (ADC) (95% CI) (N = 56,577)	0.62 (0.61, 0.63)	0.64 (0.63, 0.64)
Disease-specific survival	1-year disease-specific survival rate (NEN) (95% CI) (N = 5128)	0.96 (0.95, 0.97)	0.94 (0.93, 0.95)
	5-years disease-specific-survival rate (NEN) (95% CI) (N = 5128)	0.93 (0.91, 0.94)	0.90 (0.89, 0.91)
	1-year disease-specific-survival rate (ADC) (95% CI) (N = 56,577)	0.90 (0.90, 0.91)	0.91(0.91, 0.91)
	5-years disease-specific-survival rate (ADC) (95% CI) (N = 56,577)	0.64 (0.63, 0.65)	0.66 (0.66, 0.67)

**Table 4:** Age-specific OS and DSS rates between young-onset (<50) vs. average-onset (≥50) colorectal NENs at 1 Year and 5 Years.

Age Group (Years)	1-year Overall Survival Rate (NEN) (95% CI) (N = 25,985)	5-years Overall Survival Rate (NEN) (95% CI) (N = 25,985)	1-year Disease-Specific Survival Rate (NEN) (95% CI) (N = 25,985)	5-years Disease-Specific Survival Rate (NEN) (95% CI) (N = 25,985)
<50	0.94 (0.94, 0.95)	0.89 (0.88, 0.89)	0.95 (0.95, 0.96)	0.92 (0.92, 0.92)
≥50	0.80 (0.80, 0.81)	0.65 (0.64, 0.66)	0.85 (0.84, 0.85)	0.76 (0.75, 0.77)

## Data Availability

All the data used for this study were from the publicly available SEER database.

## Abbreviations

YOAD	Young-onset adenocarcinoma
YONEN	Young-onset neuroendocrine neoplasms
SEER	Surveillance, Epidemiology, and End Results
OS	Overall survival
DSS	Disease-specific survival
CRC	Colorectal cancer
YOCRC	Young-Onset Colorectal Cancer
GEP-NENs	Gastroenteropancreatic neuroendocrine neoplasms
NCI	National Cancer Institute
RUCC	Rural/Urban Continuum Code
AJCC	American Joint Committee on Cancer
HR	Hazard Ratio
CI	Confidence Intervals
AYA	Adolescent and young adult
MiNEN	Mixed neuroendocrine non-neuroendocrine neoplasms
MANEC	Mixed adenoneuroendocrine carcinoma
AYA	Adolescents and young adults

## Appendix A

**Table A1:** Factors affecting overall survival in YONEN (N = 2636).

Variable	Reference Group	Estimate (95% CL)	*p*-Value
**Age**			
40–49	20–39	1.14 (0.89, 1.46)	0.297
**Gender**			
Female	Male	0.87 (0.70, 1.08)	0.204
**Grade**			
II	I	1.41 (0.82, 2.43)	0.211
III	I	11.88 (8.16, 17.29)	<0.001
Undifferentiated	I	11.23 (7.37, 17.12)	<0.001
Not Reported	I	2.13 (1.48, 3.08)	<0.001
**Race**			
Asian	White	0.96 (0.65, 1.41)	0.842
Black	White	1.20 (0.91, 1.58)	0.203
Hispanic	White	0.67 (0.47, 0.95)	0.024
Native American	White	1.45 (0.53, 3.94)	0.471
**Site**			
Rectosigmoid	Colon	0.82 (0.47, 1.41)	0.468
Rectum	Colon	0.63 (0.45, 0.89)	0.008
**Stage**			
I	0	1.21 (0.17, 8.90)	0.849
II	0	1.32 (0.16, 10.58)	0.794
III	0	3.00 (0.40, 22.35)	0.283
IV	0	7.86 (1.07, 57.64)	0.043
Not Reported	0	1.75 (0.24, 12.70)	0.582
**Surgical Procedure**			
Colectomy	None	0.58 (0.40, 0.84)	0.003
Local Procedure/Wedge Resection	None	0.33 (0.24, 0.45)	<0.001
Total Proctectomy	None	0.31 (0.13, 0.71)	0.006
Total Proctocolectomy	None	0.42 (0.13, 1.36)	0.150
**Year of Diagnosis**			
2008–2011	2004–2007	0.92 (0.67, 1.26)	0.605
2012–2015	2004–2007	1.17 (0.83, 1.64)	0.371
2016–2019	2004–2007	1.33 (0.91, 1.95)	0.140

**Table A2:** Factors affecting disease-specific survival in YONEN (N = 2636).

Variable	Reference Group	Estimate (95% CL)	*p*-Value
**Age**			
40–49	20–39	1.16 (0.87, 1.54)	0.317
**Gender**			
Female	Male	0.78 (0.61, 1.00)	0.052
**Grade**			
II	I	2.57 (1.33, 4.99)	0.005
III	I	29.05 (18.12, 46.56)	<0.001
Undifferentiated	I	31.49 (18.96, 52.32)	<0.001
Not Reported	I	2.17 (1.33, 3.53)	0.002
**Race**			
Asian	White	1.15 (0.74, 1.78)	0.546
Black	White	1.02 (0.73, 1.43)	0.905
Hispanic	White	0.76 (0.52, 1.11)	0.155
Native American	White	0.49 (0.07, 3.57)	0.484
**Site**			
Rectosigmoid	Colon	1.00 (0.56, 1.77)	0.995
Rectum	Colon	0.45 (0.31, 0.65)	<0.001
**Surgical Procedure**			
Colectomy	None	0.40 (0.27, 0.58)	<0.001
Local Procedure/Wedge Resection	None	0.15 (0.10, 0.23)	<0.001
Procedure Not Reported	None	0.35 (0.05, 2.50)	0.294
Total Proctectomy	None	0.44 (0.19, 1.03)	0.059
Total Proctocolectomy	None	0.57 (0.18, 1.83)	0.342
**Year of Diagnosis**			
2008–2011	2004–2007	0.99 (0.69, 1.41)	0.954
2012–2015	2004–2007	1.04 (0.72, 1.51)	0.816
2016–2019	2004–2007	0.77 (0.51, 1.17)	0.220

**Table A3:** Restricted mean survival time model of OS in YONEN.

Variable	Estimate (95% CL)	*p*-Value
**Age**		
40–49	−0.017 (−0.042, 0.0085)	0.19
**Gender**		
Female	0.0095 (−0.017, 0.036)	0.49
**Grade**		
II	0.0094 (−0.042, 0.061)	0.72
III	−1.20 (−1.65, −0.75)	<0.001
Undifferentiated	−1.65 (−2.5, −0.79)	<0.001
Not Reported	−0.016 (−0.05, 0.018)	0.35
**Race**		
Asian	0.006 (−0.030, 0.0414)	0.74
Black	−0.028 (−0.068, 0.013)	0.19
Hispanic	0.042 (0.009, 0.074)	0.013
Native American	−0.034 (−0.15, 0.082)	0.56
**Site**		
Rectosigmoid	−0.0088 (−0.12, 0.10)	0.88
Rectum	0.036 (−0.046, 0.12)	0.39
**Surgical Procedure**		
Colectomy	0.16 (0.018, 0.29)	0.027
Local Procedure/Wedge Resection	0.13 (0.066, 0.20)	<0.001
Procedure Not Reported	0.11 (−0.16, 0.37)	0.43
Total Proctectomy	0.16 (−0.064, 0.38)	0.16
Total Proctocolectomy	0.23 (−0.0014, 0.45)	0.052
**Year of Diagnosis**		
2008–2011	0.02 (−0.039, 0.078)	0.51
2012–2015	0.015 (−0.044, 0.075)	0.61
2016–2019	−0.014 (−0.066, 0.038)	0.60

**Table A4:** Restricted mean survival time model of DSS in YONEN.

Variable	Estimate (95% CL)	*p*-Value
**Age**		
40–49	−0.01 (−0.03, 0.008)	0.26
**Gender**		
Female	−0.0043 (−0.023, 0.015)	0.66
**Grade**		
II	0.011 (−0.02, 0.042)	0.49
III	−0.005 (−0.047, 0.04)	0.83
Undifferentiated	−0.006 (−0.06, 0.04)	0.81
Not Reported	−0.0001 (−0.02, 0.02)	0.99
**Race**		
Asian	0.0065 (−0.02, 0.033)	0.62
Black	−0.024 (−0.054, 0.006)	0.12
Hispanic	0.024 (0.004, 0.04)	0.016
Native American	−0.053 (−0.17, 0.061)	0.36
**Site**		
Rectosigmoid	0.049 (−0.007, 0.11)	0.086
Rectum	0.022 (−0.029, 0.073)	0.39
**Surgical Procedure**		
Colectomy	0.014 (−0.05, 0.077)	0.68
Local Procedure/Wedge Resection	0.034 (−0.0023, 0.071)	0.067
Procedure Not Reported	−0.038 (−0.27, 0.2)	0.75
Total Proctectomy	0.048 (0.015, 0.082)	0.0048
Total Proctocolectomy	0.068 (0.026, 0.11)	0.0015
**Year of Diagnosis**		
2008–2011	−0.004 (−0.044, 0.037)	0.85
2012–2015	−0.011 (−0.05, 0.027)	0.56
2016–2019	−0.006 (−0.04, 0.028)	0.74
